# Combining Steam and Flue Gas as a Strategy to Support Energy Efficiency: A Comprehensive Review of the Associated Mechanisms

**DOI:** 10.1021/acsomega.3c09889

**Published:** 2024-03-27

**Authors:** Romel Pérez, Laura Osma, Hugo Alejandro García Duarte

**Affiliations:** † Ecopetrol S.A., Bucaramanga 681004, Colombia; ‡ Soluciones Inmediatas, Piedecuesta 681011, Colombia; § University of Calgary, Calgary, Alberta T2N 1N4, Canada

## Abstract

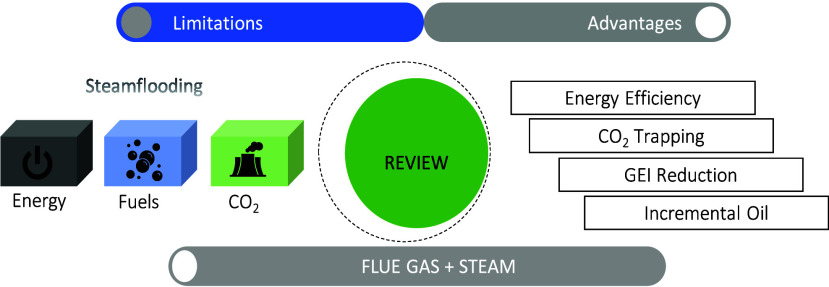

Conventional steam injection projects have long been an iconic process in the development of heavy oil reserves; nevertheless, they face significant challenges in terms of energy efficiency, environmental compliance, and economic viability. Factors such as oil price fluctuations, the imperative for an energy transition, and the push to reduce carbon footprints are hindering new or ongoing implementations of traditional steam injection technologies. In response to these challenges, hybrid methods, such as the combination of steam and flue gas, are emerging as an opportunity to optimize thermal processes to improve oil recovery, energy efficiency, and environmental sustainability and extend reservoir productivity life. Steam injection enhances oil recovery by reducing the viscosity of crude oil, improving oil mobility and facilitating its extraction. The utilization of flue gas in steam injection processes has a significant impact on oil recovery and energy efficiency, leveraging industrial byproducts. This not only lowers operating costs but also reduces environmental emissions, aligned with energy transition trends. Incorporating the flue gas into a steam-based process in heavy oil reservoirs has emerged as a promising thermally enhanced oil recovery strategy. This work presents a comprehensive review based on experimental, numerical, and field studies of hybrid steam and flue gas technology as an EOR process. The main recovery mechanisms associated with the process are analyzed. In addition, the laboratory equipment required for experimental evaluations is presented, and reservoir modeling, kinetic and compositional effects on reservoir fluids, and the reduction in heat losses in the steam injection process are discussed. Furthermore, field implementations are reviewed to evaluate lessons learned and experiences on an operative scale. The combination of steam and flue gas represents an opportunity for carbon utilization and geological carbon sequestration. This dual functionality underscores its potential to enhance oil recovery and address carbon-related environmental concerns.

## 1 Introduction

The use of steam as an enhanced recovery method in heavy oil reservoirs, in its various forms, after some time, has inefficiencies associated with the maturity of the process. Among the problems are the reduction in vertical sweep efficiency due to steam overriding, steam channeling leaving unswept zones with high oil saturation, and the early breakthrough of steam into producing wells.^1,2^ Additionally, low energy efficiencies can occur due to increased steam injection volumes to mitigate the effects of heat losses and the growth of the hot water zone due to steam condensation. Reported studies for both continuous and cyclic steam injection evaluations at maturity stages indicate steam-to-oil ratio (SOR) values^3,4^ of between 8 and 13, respectively, clearly impact the economics.^5,6^

Another relevant aspect is the environmental impact that the process itself causes; taking into account that (on average) a conventional 50 MMBTU steam generator (OTSG) fueled with natural gas emits between 70 and 72 tons of carbon dioxide into the atmosphere^3,7^ every day a greenhouse gas (GHG) identified as one of those responsible for global warming (European Commission-Energy, Climate Change, and Environment).

Under this scenario, the conventional use of steam for heavy crude oil recovery requires alternatives to overcome production, energy, and environmental challenges. In this sense, steam-based hybrid methods, involve the inclusion of some additive to the thermal process, such as solvents,^8,9^ chemical compounds^10,11^ or gases,^12^ representing viable options for increasing the recovery factor,^13^ optimizing energy efficiency, and mitigating environmental impact.^14^

**Figure 1:**
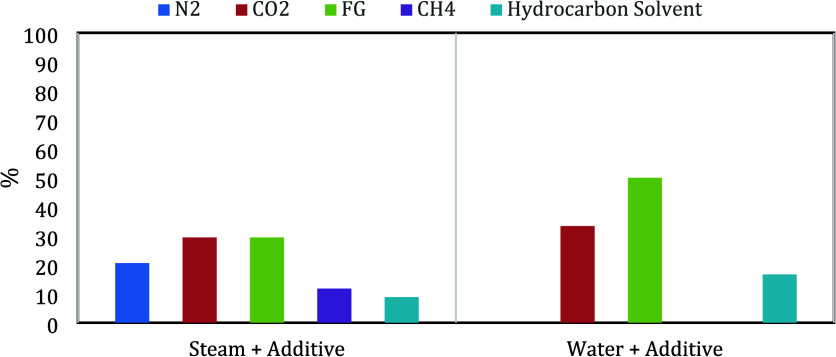
Additives used in water injection (vaporized and liquid)

Hybrid injection of steam and gases (e.g., N_2_, CO_2_, natural gas, flue gas) as a thermal recovery process has been studied since the 1980s.^15^ The intention to add an additional component to the steam arises as a possible optimization of the base process, taking into account the increase of the recovery factor and improvements in energy efficiency by replacing steam volumes with the added gases.^16,17^ The Figure 1 shows the fluids studied as additives in steam and water injection processes, taking a sample of 36 laboratory studies from the 1980s to the present.

For the thermal process, the focus is on flue gas and CO_2_, probably for two main reasons: the intention to replace a volume of steam by using flue gas and to combine the additional benefits that carbon dioxide injection could bring if solubility conditions are achieved in the crude oil.^18−21^ Flue gas was absent in the investigations involving methane and steam injection. However, carbon dioxide was included to verify which of the two components had the greatest effect on improving properties such as viscosity, density, and swelling of the crude oil when solubility conditions were reached.^22,23^ On the other hand, studies with steam, flue gas, and some hydrocarbon solvents, aim to reduce the viscosity of crude oil before steam and gas injection.^24−26^

Regarding the injection of water and some additive, the objective in all studies was to inject GHG: flue gas^27−29^ or CO_2_ only. The involvement of some hydrocarbon solvents in the CO_2_ injection stream, as with the steam processes, was conditional on studying whether it improved the solubility conditions of the CO_2_ in the crude oil.

**Figure 2:**
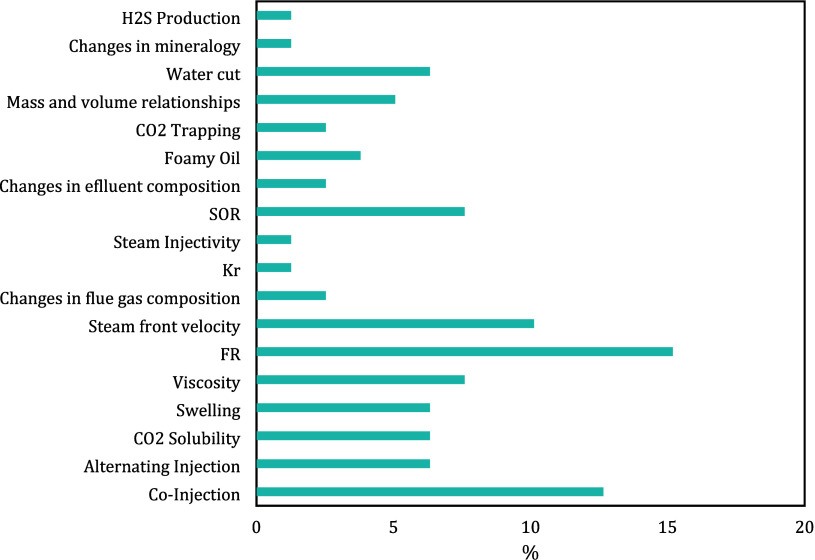
Variables identified in the experimental analyses of steam and flue gas

Figure 2 shows the variables studied in each of the experiments that involve the use of flue gas as an additive in the steam injection processes.

Concerning the steam injection scheme with the different gases used as additives (preinjection, co-injection, or postinjection), it varies depending on the geological, petrophysical, and fluid characteristics of each reservoir.^30^ For example, for a typical field in the Middle Magdalena Valley in Colombia, the appropriate scheme for cyclic injection of steam and nitrogen was coinjection, preceded and followed by 1 day of N_2_-only injection^12^ however, finding an optimal scheme is part of an integrated reservoir engineering evaluation.

**Figure 3:**
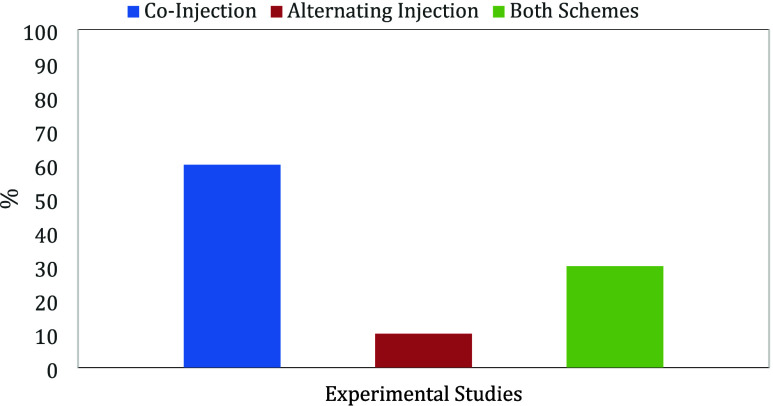
Injection schemes used in the injection of flue gas and steam (experimental and field experiences).

Figure 3 shows a quantitative percentage analysis of the injection schemes used in the different studies involving flue gas and steam injections at the experimental level.

Displacement efficiency is the variable most studied, followed by the type of injection scheme; the changes associated with the mineralogy as a result of the interactions of CO_2_ with the different minerals have not raised much interest, despite the fact that this phenomenon constitutes the most efficient mechanism for CO_2_ trapping; the change in the end points of the relative permeability curves as a result of the increase in temperature and the injection of flue gas has not generated further consideration either; the only study of this type was recently published.^31^ Finally, variables such as compositional changes of the effluents and production of H_2_S and other gases have been few analyzed.

In particular, the emphasis in this work is on evaluating experiences and phenomena associated with the use of flue gas to optimize steam injection processes. In this sense, Flue Gas (a mixture of compounds, where CO_2_ is found in a major proportion^32,33^ between 10 and 15% and 80–85% of N_2_, there are other associated components such as water, oxygen, carbon monoxide, nitrogen oxides, and sulfur oxides, among others; these are called impurities, and their presence will depend on the fuel used for its generation and the type of generator used.^34,35^

## 2 Mechanisms Associated with the Hybrid Flue Gas Steam Process

The main recovery mechanisms of the hybrid process of steam and flue gas are the combination of those associated with both steam injection and the injection of noncondensable or condensable gases (depending on the pressure and temperature characteristics under which the injection takes place), among them:

- Reduction in crude oil viscosity due to heat transfer and CO_2_ solubilization in the oil (under proper conditions^32,33^). Viscosity reductions of up to 70% are reported when CO_2_ is solubilized in crude oil^33^ associated with the increase in oil volume.
- Decreased heat losses and expansion of the heating zone due to the low thermal conductivity of N_2_ and the difference in densities. Such gas tends to be positioned in the upper strata of the formation contributing to the decrease of heat losses and favoring the increase of the heating zone.^33,36^
- Increased reservoir pressure due to the nitrogen content, due to its low solubility in oil, low compressibility, and low-pressure conditions (typically handled in most of these types of processes), supplies energy to the formation^37^
- Compositional change associated aquathermolysis.

The main reservoir properties affected by the hybrid steam and flue gas processes are discussed below.

### 2.1 Gas Solubility, Oil Viscosity, and Oil Swelling

**Figure 4:**
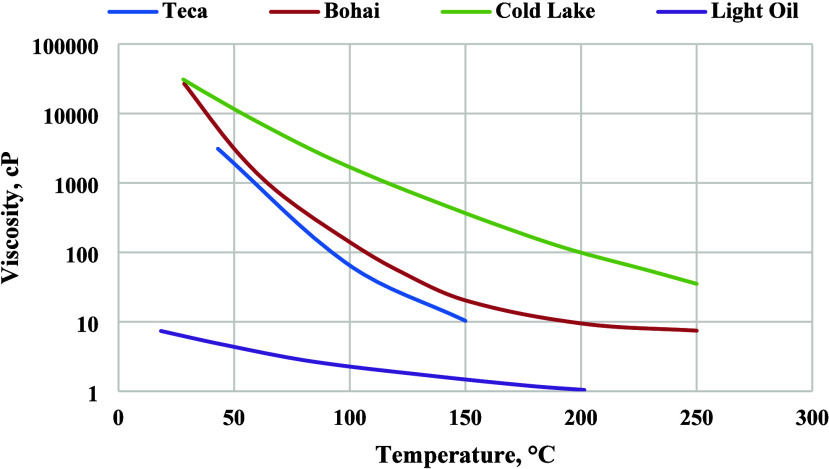
Oil viscosity vs temperature.

One of the main recovery mechanisms associated with steam injection is the reduction in crude oil viscosity combined with steam-distillation and thermal expansion of the crude oil.^38^Figure 4 shows how the heavy oil viscosity decreases by orders of magnitude with a small increase in the temperature.

In the case of heavy oil, the hybrid process of flue gas with steam is carried out in certain cases under conditions where minimum miscibility pressures are not reached; however, due to the thermodynamic conditions of the system, partial dissolution of carbon dioxide in the crude oil may occur, further reducing the viscosity and increasing the volume of the crude oil, known as oil swelling.^39^ In this sense, it is possible to achieve viscosity reductions of up to 90% under CO_2_ injection conditions in immiscible conditions.^40^

**Figure 5:**
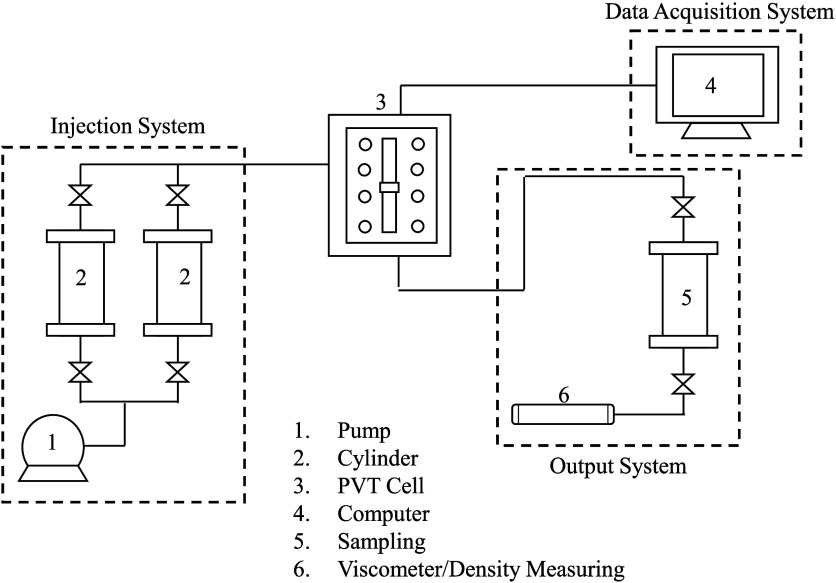
PVT assembly.

Most of the studies related to experimental analyses of the hybrid process under immiscible conditions have focused on evaluating the solubility of gases in the crude oil (Rs) through measurements of certain properties such as Rs, oil viscosity, and oil swelling at different pressures and temperatures in two-phase systems (oil–gas). The thermal effect of steam is represented by temperature variations, and possible interactions with water have yet to be discussed in depth. The typical laboratory evaluation involves a PVT assembly with a structure similar to that shown in Figure 5: a two-phase PVT cell, an injection system consisting of a pump and cylinders to contain the fluids (gas and crude oil), a data acquisition system and an outlet consisting mainly of a high-pressure cylinder and containers to collect liquid and gaseous samples for subsequent density and viscosity measurements.

The PVT results reported on the solubility of flue gas in crude oil indicate that this property improves with increasing pressure and decreases with temperature, the latter because the kinetic energy of the gas increases, causing the gas molecules to tend to escape from the solution. The greater the mass of CO_2_ present in the flue gas composition that is solubilized in the crude oil (either by increasing the concentration^27^ or by increasing the injection pressure) or by adding a solvent that acts as an auxiliary process,^41^ the greater the effect in terms of reducing the oil viscosity and oil swelling.

Taking into account the complexity of the requirements to carry out experimental studies and to know the phase behavior of crude-gas mixtures, in particular of the CO_2_-crude mixture (some limitations already described in previous sections), as an alternative, there are several applicable correlations in the literature, knowing certain properties that lead to reliable estimates regarding the solubility of the gas, changes in viscosity, density, and swelling of the crude oil. Tables 1 and 2 summarize some of the most used correlations.

**Table 1:** PVT Correlations Applied to Estimate CO_2_ and N_2_ Solubility in Heavy Oil

Mehrotra and Svrcek,^42^ (1982) CO_2_ Solubility	Mehrotra and Svrcek,^42^ (1982) N_2_ Solubility
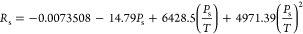 1	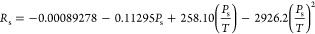 2
where, *R*_s_, CO_2_ solubility in crude oil	where, *R*_s_, N_2_ Solubility in crude oil
*T*, reservoir temperature (K)	*T*, reservoir temperature (K)
*P*_s,_ saturation pressure (MPa)	*P*_s,_ saturation pressure (MPa)
Chung^43^ et al. (1988) CO_2_ solubility	Rostami,^44^ (2017) CO_2_ solubility
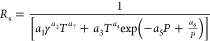 3	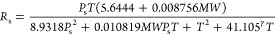 4
where, *R*_s_, CO_2_ solubility in crude oil	where, *R*_s_, CO_2_ solubility in crude oil (molar fraction)
*T*, reservoir temperature (°F)	*T*, reservoir temperature (°C)
*P*_,_ saturation pressure (Psia)	*P*_s,_ saturation pressure (MPa)
γ, specific gravity	MW, molecular weight (g/mol)
*a*_1_ = 0.4934 × 10^–2^, *a*_2_ = 4.0928, *a*_3_ = 0.571 × 10^–6^, *a*_4_ = 1.6428, *a*_5_ = 0.6723 × 10^–3^, *a*_6_ = 781.334, *a*_7_ = −0.2499; application range // *P* < 20.7 MPa and API [10–20°]	γ, specific gravity
	R^2^ = 0.9860 = Coefficient of determination

**Table 2:** PVT Correlations Applied to Estimate Oil Viscosity Saturated with CO_2_ or N_2_

Mehrotra and Svrcek,^42^ (1982) Oil Viscosity Saturated with CO_2_	Mehrotra and Svrcek,^42^ (1982) Oil Viscosity Saturated with N_2_
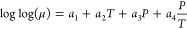 5	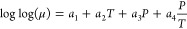 6
where, μ, oil viscosity	where, μ, oil viscosity
*T*, reservoir temperature (°C)	*T*, reservoir temperature (°C)
*P*, pressure (MPa)	*P*, pressure (MPa)
*a*_1_ = 0.815991, *a*_2_ = −0.0044495, *a*_3_ = 0.076639, *a*_4_ = −34.5133	*a*_1_ = 0.804065, *a*_2_ = −0.00442099, *a*_3_ = −0.00589803, *a*_4_ = 1.86224

Lederer^43^ (1933) Oil Viscosity Saturated with CO_2_	Chung^43^ et al. (1988) Oil Viscosity Saturated with CO_2_
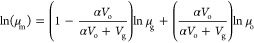 7	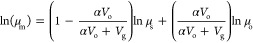 8
where, μ_m_, oil viscosity saturated with CO_2_	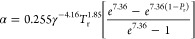 9
α, adjustment parameter (between 0 and 1)	where, μ_μ_, oil viscosity saturated with CO_2_
μ_g_, CO_2_ viscosity	μ_g_, CO_2_ viscosity
μ_o_, oil dead viscosity	μ_o_, oil dead viscosity
*V*_g_, CO_2_ volume fraction	*V*_g_, CO_2_ volume fraction
*V*_o_, oil volume fraction	*V*_o_, oil volume fraction
α, adjustment parameter	*T*_r_, reduced temperature
	*P*_r_, reduced pressure
	average absolute deviations = 3.5%

### 2.2 Reservoir Pressure and Heat Losses

Under immiscible injection conditions, flue gas, nitrogen, or methane provides additional recovery mechanisms that increase reservoir productivity. Some experiences reported in the literature indicate that the incorporation of gases in both continuous and cyclic steam injection schemes leads to a reduction in heat losses to the overburden and into the well.^45^ It also increases the size of the heated zone, and the gases provide additional energy to the process.^6^ It has been shown that when N_2_ is injected for hybrid steam processes, the low conductivity of this gas allows heat losses to be reduced by 2–5% and steam quality to be improved^46^ by 5–6%. Its density and low thermal conductivity, in contrast to those of water and oil, allow it to act as an insulator during steam injection, being in the upper layers and preventing high heat losses. Some relevant references are given below:

- Jianfang,^45^ 2012, using numerical simulation, demonstrated that cyclic steam injection and nitrogen in heavy oil and using horizontal wells increased the heating efficiency, reporting improved enthalpies of 7.3% and increases between 0.5 and 1 °C of reservoir temperature after nitrogen injection.
- Liu^33^ et al., 2012, demonstrated through numerical simulation that injection of steam and noncondensable gases in horizontal wells increased the size of the steam chamber by up to 2 times and promoted an increase in reservoir pressure between 0.2 and 2 MPa.
- Wenjiang,^47^ Xu et al., 2014, Combining experimental studies in linear physical models, numerical simulations, and the implementation of a field pilot test (12 wells were stimulated), demonstrated that the coinjection of noncondensable gases and steam in offshore heavy oil wells is beneficial in preventing heat loss, as gases accumulate in areas far from the wells, forming a kind of insulating layer.
- Liu^38^ et al., 2020, through experiments in a linear displacement physical model, found that the injection of noncondensable gases and steam in horizontal heavy crude oil wells improved the temperature and pressure distribution in the reservoir, reporting a reduction in the drop of the variables between 12% and 15% compared to steam-only injection.
- Moussa^48^ et al., 2018, also demonstrated the positive effect of nitrogen on reservoir pressure, reducing energy losses and increasing reservoir temperature. Through a numerical simulation study, it proposes the generation of nitrogen and steam in situ through exothermic reactions between water injected under liquid conditions and certain highly exothermic reagents and contrasts the results with a conventional steam injection scenario.

There are numerous numerical simulation studies, experimental results, and multiple pilots that also account for the positive effect of noncondensable gases and steam for SAGD applications, evidencing the same principle: the accumulation of gases at the top of the formation, which acts as an insulating layer, and in this way the heating efficiency will increase. Additionally, the gases also provide energy to the process and will reduce the steam requirements, since by reducing the temperature of the upper part of the chamber to values below the steam saturation temperature, the energy supply to maintain the chamber is lower.^36,49,50^

### 2.3 Aquathermolysis and Compositional Changes in Crude Oil

It has been reported that the injected gas causes an alteration in the composition of the produced fluids and residual crude oil. Through mass transfer and distillation mechanisms, the gas phase extracts light and intermediate components from heavy crude oil, causing possible asphaltene precipitation and deposition in the reservoir and, in certain cases, creating unfavorable mobility conditions for the remaining crude oil. Some relevant studies are

- Dong^27^ and Huang, 2002, carried out PVT studies to evaluate the solubility of three different types of flue gas in Canadian heavy crude oil (type Senlac), the compositional analyses of the gas phase showed that the little methane solubilized in the base crude oil of the experiments was extracted by the injected gas and replaced by the CO_2_ and N_2_ of the flue gas.
- Wang^51^ et al., 2015, found through displacement tests that excess nitrogen coinjected with steam in Bohai heavy crude oil decreased the displacement efficiency of the process. Without performing compositional analysis, it was suggested that the excess injected gas was extracted components from the crude oil, and this was the cause for the gas prematurely breaking into the production, so an optimal gas-steam ratio needed to be estimated.
- Wang^28^ et al., 2017, through rock-fluid displacement with flue gas and steam using dead oil from the Liaohe field, analyzed the composition of the gas produced and the results showed that this changed over time, before the gas was channeled, the percentage of CO_2_ was lower than that injected, indicating that dissolution and diffusion phenomena occurred, however, after the gas breakthrough, the amount of CO_2_ increased, but never to the proportion injected, and in addition, the crude oil produced had characteristics of foamy oil, a fluid with microbubbles and improved mobility. Few steam-based hybrid studies report on the effect of the extraction of light and intermediate components on the oil phase, either by SARA or other compositional analyses. It is reported the capability of CO_2_ or N_2_ to extract components through gas chromatography of the expansions in PVT experiments.^37^
- Songyan^52^ et al., 2017, found in the effluents from displacement tests with steam and flue gas, for Shengli heavy oil that the content and molecular weight of the asphaltene fraction began to decrease (reaching maximum reductions up to 30% and 12%, respectively), after the breakthrough of the injected gas, as a result of the carryover of light components. The saturates and resins fractions were also affected and increased by 2.5% and 0.6%, respectively. The analyses of the residual crude oil in the corepack showed that the fluid, unlike the effluents and the base crude oil of the experiments, presented deteriorated characteristics (high asphaltene fractions), which would most likely cause problems of damage to the reservoir.
- Wei,^53^ et al., 2022, analyzed the volumetric and mass flue gas-vapor ratios on the SARA fractions of crude oil produced in displacements and found that as the flue gas content increased, the effect of distillation increased and the content of light components in the residual crude oil decreased.

The phenomena of in situ gas generation in steam-based hybrid processes have been somewhat studied and are reported. In this sense, Pérez^54^ et al., 2022, obtained the production of H_2_, CO, and a considerable excess of CO_2_ in their hybrid displacements with steam and flue gas for Colombian crude oil, suggesting the influence of aquathermolysis, without detailing the effect of N_2_ or injected CO_2_ on the phenomenon. For conventional displacements with steam, there is evidence from experimental studies reporting the production of gases such as CO_2_, CO, H_2_, H_2_S, CH_4_, and other light compounds,^55^ but there is no consensus on their origin: the causes are located between aquathermolysis phenomena.

The aquathermolysis phenomenon involves a series of chemical reactions between the organosulfur compounds of crude oil, water, and certain minerals in the rock acting as natural catalysts in a temperature^56^ range between 250 and 300 °C. The sequence begins with the breakdown of the organosulfur compounds of the crude oil through hydrolysis reactions that produce^57^ H_2_S.
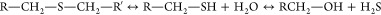
10This is followed by a reorganization of the unsaturated alcohols and the formation of aldehydes, which then give way to the formation of carbon monoxide.^58^
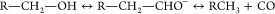
11Carbon monoxide undergoes water–gas shifting reactions forming hydrogen and carbon dioxide
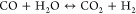
12

However, the volume of CO_2_ generated and reported is almost always greater than the volume of H_2_S and the reactions proposed by Hyne^56^ stoichiometrically indicate that the amount of carbon dioxide is equal to that of hydrogen sulfide, so it is inferred that the origin of CO_2_ cannot be solely attributable to the phenomena of aquathermolysis. In this sense, Thimm,^58^ 2014, proposes that the decarboxylation of crude oil is a source of CO_2_ production:
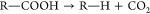
13

Similarly, the interactions between water and certain minerals present in the rock,^56^ the hydrolysis of carbonates from acid solutions at high temperatures^57^ also represent a source of CO_2_.
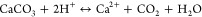
14
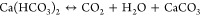
15

Several thermogravimetric studies have been carried out to measure the oxidative behavior of different crude oils,^59^ as well as tests in reactors at high pressure and high temperature are also well documented to establish fluid–fluid interactions between crude oil and noncondensable gases at high temperatures and their effect on the SARA fractions, the composition of the crude oil and the estimation of kinetic schemes, mainly focused on processes such as air injection or in situ combustion.

In this sense, Chen^3^ et al., 2018, evaluated the fluid–fluid interaction between heavy Liaohe crude oil from China and different noncondensable gases (N_2_, CO_2_, air, flue gas) through reactor tests at high pressure and high temperature. They found that by increasing the temperature for the case of flue gas (3% O_2_, 11.93% CO_2_, and 85.07% N_2_), carbon dioxide production increased due to oxidation reactions of the carbon groups due to the presence of O_2_. The SARA analyses showed an increase in the fraction of asphaltenes and resins as a result of oxidative thermal degradation produced by the oxygen present in the flue gas; additionally, the fraction of saturates and aromatics decreased due to the transformation of a small fraction of crude oil into gas. However, published experimental evidence evaluating the effect of CO_2_ or N_2_ from flue gas on aquathermolysis reactions is not widespread in the literature.

In a study by Mecón^60^ et al., 2022, through tests in a batch-type microreactor with steam-flue gas-crude and different mineralogy, it was evident that the presence of certain minerals and the CO_2_ of the flue gas promote polymerization reactions, a chemical subprocess associated with aquathermolysis, where by the action of temperature generates the breaking of complex chains and the unstable free radicals intertwine, forming more complex and heavier compounds, thus increasing the initial viscosity of the crude oil.^61^

## 3 Flue Gas Injection and CO_2_ Storage

It is important to mention that the main objective of studies involving the injection of combustion gases together with steam is to increase the recovery factor and not to store or capture the injected^29^ CO_2_, however, based on the growing global interest in the reduction of GHG and its impact on reducing global warming, the steam-based hybrid process represents an opportunity to couple oil recovery with gas storage (EOR-CCUS). The benefits in terms of use or storage will be affected by the geological, structural, petrophysical, and fluid characteristics of each field, and determining the feasibility of field projects. There are well-known mechanisms of carbon dioxide trapping that are discussed below:

### Structural and Stratigraphic Trapping

This is related to the type of geological structure in which the process is to be carried out; this requires a physical trap consisting of an anticline or sealing fault. In this mechanism, the reservoir basically acts as a retention barrier to the injected gas. Depleted or mature reservoirs are usually a target for CO_2_ storage because they have all the infrastructure for injection and a detailed geological description of the subsurface in a way that ensures long-term trapping of the gas.^62^ The storage capacity will depend on characteristics such as porosity, permeability, and depth, as it is desired to keep the CO_2_ in a supercritical state, thus reducing its volume and increasing the storage capacity.^63^

### Hydrodynamic Trapping

Depending on the case, the injected CO_2_ migrates toward saline aquifers and is trapped there by the natural water flow. The amount of CO_2_ trapped is estimated based on the reservoir dip, the velocity of the water flow, and the direction of the aquifer; however, the transit time of CO_2_ dissolved in water can be thousands of years, for example, which is considered safe for long-term storage.^64^

### Residual Trapping

Also known as capillary trapping, it is related to the ability of the pore space to trap gas. Various experimental and numerical studies report that up to 47% of the injected gas can be trapped by this phenomenon,^65^ making it particularly important and considered the most efficient mechanism for storage, especially when EOR processes are involved. When the gas is injected, it displaces the fluids of the reservoir (brine or hydrocarbons) and, due to differences in pressure and density, it moves to the top of the formations; once the injection stops, a redistribution of the native fluids of the reservoir occurs in the porous medium, displacing the gas bubbles and small capillary-sized droplets are isolated and trapped in the pore space.^66,67^

The capability of capillary trapping is determined by three factors: wettability (a strongly water-wet formation has a greater degree of trapping), the relationship between pore and throat size (trapping is favored when throat size is smaller relative to pore size), and connectivity between pores (well-connected systems are less prone to trapping^66^).

Residual gas saturation (Sgr) is the parameter that allows quantification of the volume of gas trapped in the pore space due to the action of capillary forces.^68^ The Sgr can be estimated using neutron, sonic or resistivity logs,^69^ pilot tests with partition tracers,^70^ digital rock analysis^71^ and numerical models such as those proposed by Naar and Henderson,^72^ 1961, Aissaoui,^73^ 1983, Jerauld,^74^ 1997, and Land,^75^ 1968, among others.

The analytical models developed are based on the concept of relating the initial and residual saturation of the nonwetting phase, this relationship is known as IR (for initial-residual) and each model allows different degrees of freedom to fit experimental or field data.^66^ The IR model developed by Land,^75^ shown below, was developed to attempt to fit experimental data of permeabilities relative to an imbibition process in two-phase and three-phase flows in porous media:^75^
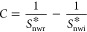
16

Where *S*_nwr_^*^ and *S*_nwi_^*^ are the normalized saturations (residual and initial, respectively) of the nonwetting phase and *C* is a constant, known as the trapping coefficient, which allows the adjustment of the experimental data; this constant can take different values depending on the type of rock.

Residual trapping has been used in recent years as a way to parametrize hysteresis models in relative permeabilities and capillary pressures.^66^ It is assumed that the differences in relative permeabilities and capillary pressures between each imbibition and drainage stage are due to the trapped saturation of the nonwetting phase.

Commercial simulators (e.g., CMG) include different options to estimate the trapping of the wetting phase but as an input for hysteresis modeling. In the case of the compositional simulator (e.g., GEM), there are 3 hysteresis models available, 2 for two-phase systems (Carlson and a self-developed model) and 1 for three-phase systems (Larsen and Skauge 1998), and depending on the case, there are the possibilities to vary the modeling of the trapping of the nonwetting phase either through the Land^75^ and Aissaoui,^73^ 1983 models or table data interpolation. The inclusion of two-phase models to represent hysteresis and gas trapping in three-phase systems is not appropriate since they assume reversibility of the relative permeability curves for a second drainage cycle after imbibition, and these assumptions have not been validated with experimental data.^76,77^ In the case of the thermal simulator (e.g., STARS), three hysteresis models are included as an option to represent the phenomenon in thermal processes (*CARLSON, *KILLOUGH, and *BBM).^78^

According to previous research,^76^ there are three specific phenomena in the estimation of trapped gas saturation at the gridblock scale in commercial simulators that can lead to errors in the calculation: (a) The land^75^ model is not capable of estimating a dynamic trapped gas saturation, and since in many grid blocks the residual saturation required by this model is not reached, the hysteresis phenomenon is misrepresented; (b) the choice of the three-phase relative permeability model also introduces associated errors, since, depending on the type of saturation function used, it may incorrectly calculate the relative permeabilities of the intermediate phase and consequently cause numerical instabilities; and (c) mass errors in trapped gas saturation, which means that if there are changes in the composition, trapped gas saturation values of zero may occur at the gridblock level due to gas dissolving in the oil phase, a phenomenon that is not consistent with what has been observed in the laboratory.

### Trapping by Solubility (in Water and Crude Oil)

CO_2_ can also be trapped in the reservoir by solubility mechanisms in crude oil or water. CO_2_ dissociates easily in water, forming a weak acid, and its dissolution capacity is directly proportional to pressure, but inversely proportional to temperature and salinity of the brine.^78^ Dissolution in the aqueous phase is represented by a reversible reaction, as shown below.
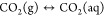
17

Where, the subscripts g and aq indicate CO_2_ in the gaseous and aqueous phases, respectively. The solubility of CO_2_ in water can be estimated by PVT experiments or by calculating the fugacity^78^ using Henry’s Law:
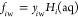
18where *f*_*i*w_ is the fugacity of component *i* in the aqueous phase, *y*_*i*w_ is the mole fraction of component *i* in the aqueous phase, and *H*_*i*_ is the Henry’s Law constant of component *i*, which can be calculated as a function of pressure at isotherms conditions:
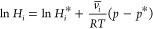
19where *H*_i_^*^ is Henry’s constant at the reference pressure and temperature, *v̅*_*i*_ is the molar partial volume of component *i* in the brine, and *R* is the universal gas constant.

For thermal processes, the mole fraction of CO_2_ in each phase under thermodynamic equilibrium conditions is modeled by the equilibrium constants. Murayri^79^ et al., 2011, propose to calculate the equilibrium constants of carbon dioxide in water for a SAGD process, using the following equation:
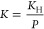
20where *K*_H_ is Henry’s law constant and *P* is the reservoir pressure. To obtain this expression, it was assumed that CO_2_ behaves as an ideal gas under the operating conditions of the pressure and temperature of the process. To calculate Henry’s constant at different temperatures, the correlation of Harvey,^80^ 1996, is used:
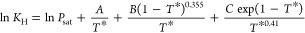
21where, *P*_sat_ is the saturation pressure of the water at the specified temperature conditions, T^*^ is the ratio between the critical and operating temperatures of the water, and *A*, *B*, and *C* are correlation parameters that vary depending on the gas used, in the case of CO_2_, they are −9.4234, 4.0087, and 10.3199, respectively. Gillis^81^ et al., 2000, suggest calculating Henry’s constant using the correlations of Carroll^82^ and Mather, 1989, for temperatures below 100 °C and Suleimenov^83^ and Krupp, 1994, for temperatures above 100 °C.
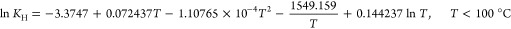
22
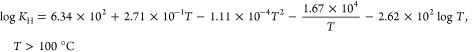
23where *T* is the operating temperature (K).

The solubility of CO_2_ in crude oil can be estimated by PVT experiments (previously discussed in section 2, such as solubility, viscosity, and swelling), by correlations, by slim tube tests (estimation of minimum miscibility pressure), or by equation of state models that calculate the mole fractions of CO_2_ in the oil or gas phase using the fugacity calculation.^84^ Ding^85^ et al., 2018, estimated that for a depleted reservoir, the maximum storage capacity due to CO_2_ solubility in oil and water was 2.82 Mt and 0.48 Mt, respectively. According to them, the parameters that most influenced solubility in oil increasing storage were pressure and high injection rates. On the other hand, solubility in water was strongly impacted by salinity and temperature. However, the objective of the study was geological storage, not hydrocarbon production.

### Mineral Trapping

This mechanism is related to solubility trapping, basically CO_2_ dissolved in water produces carbonic acid, which dissociates into hydrogen protons and bicarbonate ions according to the following model:
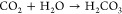
24
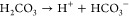
25Increased acidity in the environment promotes chemical reactions with the rock, in which there is dissolution of native minerals present in the rock, such as Ca^2+^, Mg^2+^, Fe^2+^, which react with the bicarbonate ion to form solid carbonates^85^
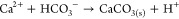
26
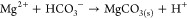
27
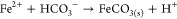
28

Mineral trapping can be estimated using analytical models such as those proposed by Ding^85^ et al., 2018, or by Xu^34^ et al., 2004, who also noted that this mechanism also varies depending on the type of rock. In the case of commercial simulators, compositional and thermal simulators usually have a special “geochemistry” module that allows modeling this type of processes.^78^

### Storage and Operational Strategies

Well location, produced gas monitoring strategies, and knowledge of the geological structure, among others, are key aspects to maximize CO_2_ storage. In addition, consideration of GOR constraints,^86^ partial completions,^87^ operational management of the BHP in producing wells, and injection rates can increase the storage capacity of CO_2_ injected into the flue gas stream. These strategies, combined with knowledge of possible trapping mechanisms, can help define specific strategies to maximize oil recovery and CO_2_ storage for the hybrid steam and flue gas process.

## 4 Field Applications

Some references have reported field cases of steam and flue gas injection in different fields worldwide and under different injection schemes, mainly in the United States of America, China, and Canada. Table 3 shows the main field applications of flue gas injection without steam and like a steam-based hybrid process.

**Table 3:** Field Case of Flue Gas Injection

Country	Field	Year	Process	Flue Gas Source
United States of America	LaCygne–Cadmus	1990	Cyclic Flue Gas Injection	Own generation/combustion chambers
United States of America	Marchand	1979	Continuous Flue Gas Injection/Miscible	
United States of America	East Edna	1998	Continuous Flue Gas Injection	
Canada	McMurray	2004	SAGD + Flue Gas Injection	
China	Bohai	2009	Cyclic Steam-Flue Gas Co-injection	Portable steam generator and Flue Gas
China	Xinjiang	2016		
China	Shengli	2020	Cyclic Steam-Flue Gas Co-injection	Capture of the gases from the steam generator

A brief description of each project is given below.

### Twin Project, Kansas^88^

This project implemented cyclic flue gas injection under immiscible conditions in the LaCygne–Cadmus field. The characteristics of the reservoir are shown in Table 4.

**Table 4:** Rock Fluid Properties, LaCygne–Cadmus Field

Property	Value
Depth	200–300 ft
Thickness	15–30 ft
Permeability	35 mD
Porosity	0.19
Temperature	78 °F
Viscosity	20–30 cP
°API	29

The flue gas supply comes from the combustion of natural gas in a conventional combustion chamber fed daily with 11,000 ft^3^/day, also allowing the use of propane. It produced 100,000 ft^3^/day of flue gas with an average composition of 13% CO_2_ and 87% N_2._ The produced gas stream was treated to remove impurities, then cooled, and condensed liquids were removed. The wells were stimulated in a 21-day injection period followed by a soak day, and the total volume injected was 350,000 ft^3^ of flue gas. Injection began in 1979 and continued until 1988, reaching production peaks of 120 barrels per week, much higher than that observed in the field prior to the start of the process. During the 10 years of the project, 43,000 barrels of crude oil were produced and the recovery factor increased by 43%.

### Caddo County Project, Oklahoma^89^

Flue gas injection was conducted under miscible conditions from 1977 to 1986 in the Marchand formation, which has permeabilities of 1 mD and fluids of 42°API. As in the Twin project, the flue gas was supplied from a combustion chamber designed to produce it at a rate of 30,000 ft^3^ per day at a pressure of 4,500 psi. A series of reactions occurred in the reservoir that produced excess N_2_, so a plant was built in 1986 to manage the excess N_2_ produced and the subsequent reinjection of the gas, also under miscible conditions. During the flue gas injection, there were serious problems of injectivity and corrosion. However, in the end it was more profitable to produce and inject nitrogen.

### East Edna Field, Oklahoma^90^

Flue gas was injected in a pattern of inverted five-spot spaced less than 10 acres, where oil production did not exceed 2 barrels per day. The objective was to increase the reservoir pressure. Reservoir characteristics are shown in Table 5.

**Table 5:** Reservoir Properties East Edna Field

Property	Value
Depth	2500 ft
Net pay	12 ft
Permeability	5–20 mD
Porosity	0.12
Temperature	78 °F
Initial Pressure	840 psi
°API	30

For this project, the flue gas was generated by an internal combustion engine fueled by natural gas produced in the field. The total volume injected was 25 MMSCF (during 1 year), representing approximately 40% of the pore volume of the reservoir. At the end of the first year of injection, rates of 12–14 BPD were achieved. However, gas production and channelization of the injected fluid increased, mechanical problems, corrosion of the equipment, and a decrease in the calorific value of the produced gas occurred, all of which led to the cancellation of the project.

### Proyecto Dover, Canada^91^

Flue gas was injected into SAGD pairs located in the McMurray formation; the characteristics of the reservoir are shown in Table 6.

**Table 6:** Dover Reservoir Properties

Property	Value
Depth	426 ft
Net pay	50–80 ft
Permeability	3–5 D
Porosity	0.32–0.35%
Temperature	44 °F
Viscosity	3 × 10^6^ cP
°API	8

To provide the injection gas, this project used a system known as an exhaust gas processor (EGP), which, like other field applications, used internal combustion engines or chambers fed with natural gas or propane. The gas produced was then treated to remove impurities and moisture. The requirements for the volume of injected steam decreased with the injection of gas as a hybrid technology.

### Multithermal Fluid Injection, China

Due to the size and weight of conventional steam generators, their installation in offshore fields is complex and limited, so China has been searching for alternatives that allow the exploitation of heavy crude oil resources offshore. To this end, they have implemented a new technique called multi-thermal fluid stimulation (MTFS), which consists of the coinjection of flue gas and steam at high temperatures and pressures.^92^ The injected fluids are produced by a portable generator, and according to the developers, it differs from the injection of steam and noncondensable gases in three points: (a) the fluids are always coinjected, (b) the composition of the gas flows with other gases in the composition (CO and CH_4_), and (c) the injection pressures and temperatures are high, which represents savings associated with the treatment and cooling of the combustion gas stream. Theoretically, each portable generator can produce 166 tons of steam at 300 °C and 4.82 × 10^4^ m^3^/day of flue gas, and the enthalpy of this combination is equivalent, to 2.6 × 10^8^ kJ according to manufacturers.^92^

### Bohai Field

This system has been applied in the NB35-2 field in the western Bohai area; the first MTFS pilot was conducted in 2009 as a cyclic injection process in more than 15 offshore horizontal wells. As of December 2018, 27 cycles have been performed, and 145 × 10^4^ m^3^ of incremental oil has been reported. Of these 15 wells, there are 4 wells where 3 consecutive stimulation cycles have been performed, and the response has been successful: each well has reached daily rates of 40–60 m^3^/day, which is two or three times the baseline. In addition, the energy efficiency has been improved by extending the duration of the cycles from 300 days to 600–800 days in the first cycle and up to 1000 days in the second cycle.

### Xinjiang Field

In 2016, the field also carried out multithermal fluid injection in 4 blocks with different heavy oils. Updated reports indicate that more than 15 wells have been intervened with high rates of incremental oil production, averaging 291 tons per well.^92^

### Shengli Project

Since 2011, this heavy crude oil field had been cyclically steam injected; in 2020, a well that had previously been subjected to 10 steam stimulation cycles was injected with the hybrid technology of steam and flue gas. A total of 1950 tons of steam at a temperature of 260 °C and 510,000 m^3^ of flue gas with a molar composition of CO_2_ equivalent to 13% were injected. Once the well was in production, an increase of 1027 tons of crude oil was obtained compared to the previous steam cycle, and in terms of CO_2_ storage, it was estimated that of the 131.1 tons injected, only 33.4 tons were produced, resulting in a CO_2_ storage of 74.5% of the carbon dioxide injected.

The source of the injected flue gas was the capture of combustion gas emissions from the field’s steam generators; for this purpose, this project used a system since natural gas was used as a fuel to generate steam, the process of cleaning and conditioning the captured flue gas was simple: removing particles as well as small amounts of hydrogen sulfide and moisture, and finally compressing it in two stages and injecting it at high pressure along with steam^93^ and the daily flue gas injection capacity was 1200 m^3^/h.

## Conclusions

1. The objective of experimental studies at the rock-fluid for the hybrid steam and flue gas technology is associated with the interest of increasing displacement efficiencies and the optimization of operational variables such as rates, volumes, and injection schemes.
2. For the fluid–fluid experiments, PVT analyzes are oriented toward measuring CO_2_ solubility conditions and their impact on properies such as viscosity, density and swelling of the crude oil, as well as basic characterizations of the original crude oil. This information will be required for the construction of robust fluid models, however, in the absence of experimental data, correlations can be used according to specific conditions.
3. The thermal insulating effect of the nitrogen, a major component of flue gas, has been widely tested and confirmed from the numerical reservoir simulation approach and with the results of multiple pilots N_2_ + steam and SAGD + noncondensable gases.
4. Some experimental evidence reported compositional changes in crude oil, associated with the extraction of light and intermediate components caused by the injection of flue gas.
5. Although the aquathermolysis reactions associated with steam injection processes has been described and widely documented, however for the hybrid process of steam and flue gas there is no strong evidence on the occurrence of the different chemical reactions linked to the aquathermolysis phenomenon that describes the volumes of gases produced and the degree of upgrading of the oil.
6. The hybrid injection of steam and flue gas represents an opportunity to combine a thermal recovery process with CO_2_ storage. There are trapping mechanisms associated, where capillary and mineral are the most efficient; however, their applicability will be subject to the particular geological, structural, petrophysical, and fluid characteristics of each reservoir.
7. According to the identified field applications of the steam and flue gas hybrid technology, it is notable that the only injection scheme implemented corresponds to the coinjection.
8. The sources of flue gas supply varied from generation from combustion chambers, generators portables that coinject steam and combustion gases and recently the capture of flue gas from conventional steam generators was reported.

## Nomenclature

CO_2_: carbon dioxide
CSS: cyclic steam stimulation
EOR: enhanced oil recovery
H_2_S: hydrogen sulfide
N_2_: nitrogen
SF: steam flooding
SG: steam generator
SOR: steam oil ratio
TC: thermocouple
tEOR: thermal enhanced oil recovery
Nm^3^: nominal cubic meter
Mt: million ton
